# Association of *HLA* class I and II genes with cutaneous leishmaniasis: a case control study from Sri Lanka and a systematic review

**DOI:** 10.1186/s12879-016-1626-8

**Published:** 2016-06-14

**Authors:** Nilakshi Samaranayake, Sumadhya D. Fernando, Nilaksha F. Neththikumara, Chaturaka Rodrigo, Nadira D. Karunaweera, Vajira H. W. Dissanayake

**Affiliations:** Department of Parasitology, Faculty of Medicine, University of Colombo, 271, Kynsey Road, Colombo, 008 Sri Lanka; Human Genetics Unit, Faculty of Medicine, University of Colombo, Colombo, Sri Lanka; Department of Clinical Medicine, Faculty of Medicine, University of Colombo, Colombo, Sri Lanka

**Keywords:** Genetic predisposition, *Leishmania donovani*, Host-parasite interactions, Immune response genes, Neglected diseases, Genetic polymorphism, Asia

## Abstract

**Background:**

The outcome of leishmaniasis is an interplay between *Leishamania* and the host. Identifying contributory host genetic factors is complicated by the variability in phenotype, ethnicity and parasite species. Leishmaniasis is caused exclusively by *L. donovani* in Sri Lanka with localized cutaneous leishmaniasis (LCL) being the predominant form. We report here an association study of human leucocyte antigen (*HLA)* class I and II genes with LCL in Sri Lanka, the first on *HLA* associations in cutaneous leishmaniasis in a South Asian population.

**Methods:**

An existing DNA repository of 200 each of patients and controls was typed for *HLA-DQ* by PCR-SSP. Next generation sequencing-based typing for *HLA-A*, *HLA*-*B* and *HLA*-*DRB1* alleles was done in a subset of 280 samples. Association tests were performed on 28,489 genotyped and imputed SNPs spanning a region of 1.4 Mb across the *HLA* genes. To compare our results with similar studies, we carried out a systematic review to document all HLA associations reported to-date for cutaneous and muco-cutaneous leishmaniasis.

**Results:**

DRB1*04 DQB1*02 (*P =* 0.03; *Pc* = 0.09), DRB1*07 DQB1*02 (*P =* 0.03; *Pc* = 0.09) haplotypes were absent in patients. B*07 (*P =* 0.007; *Pc =* 0.13; OR = 0.36; 95 % CI = 0.17–0.77) allele and DRB1*15 DQB1*06 (*P =* 0.00; *Pc* < 0.01; OR = 0.3; 95 % CI = 0.2–.0.6) haplotype were over represented in controls and DRB1*15 (*P =* 0.002; *Pc* = 0.01) allele was over represented in patients. Two SNPs (rs281864595/rs1050517) in the antigen recognition region of *HLA-B*, comprised a haplotype more frequent in controls (*P =* 0.04). The alleles identified by the systematic review to predispose or to protect from cutaneous/mucocutaneous leishmaniasis remained highly heterogeneous in different populations studied.

**Conclusions:**

Our preliminary findings suggest a role for some class I and class II *HLA* genes in determining predisposition to LCL in this population which should be corroborated with further studies. The systematic review reiterates this need, as the purported susceptibility or protection gained by certain *HLA* alleles or haplotypes has rarely been independently verified.

**Electronic supplementary material:**

The online version of this article (doi:10.1186/s12879-016-1626-8) contains supplementary material, which is available to authorized users.

## Background

Leishmaniasis is an anthropo zoonotic disease with a global incidence of approximately two million cases [1]. Caused by a protozoan parasite of the genus *Leishmania* and transmitted by the sandfly, this is a disease with a spectrum of phenotypes ranging from self-healing localized skin lesions to potentially fatal visceral disease. Classically, different species of *Leishmania* are implicated in the distinct forms of the disease.

Cutaneous disease, even if not fatal can be chronic, disfiguring and stigmatizing and bears the brunt of the disease burden with 1.5 million new cases reported every year [2]. The cutaneous form itself has a range of clinical presentations with localised cutaneous leishmaniasis (LCL) being the commonest. The severity of cutaneous disease depends on both the extent of parasite replication and the relative induction of immunopathologic responses by the host. The genetic background is considered the single most important host factor which modulates these immune responses [3–6].

The human major histocompatibility complex (MHC) gene cluster on the short arm of chromosome 6 has been central to many studies investigating host genetic factors as determinants of outcome of leishmaniasis. The classical human leukocyte antigen (*HLA*) genes of class I and II located within the MHC, encode molecules identified for their role in presentation of antigens to CD8^+^ and CD4^+^ T cells respectively. CD4^+^ T cells are the major source of interferon (IFN)-γ which controls parasite multiplication during the early phases of *Leishmania* infection [7, 8] whereas CD8^+^ T cells have been described to contribute to IFN-γ production and the differentiation of Th1 responses [9, 10]. Furthermore, localised cutaneous lesions due to *L. (V.) braziliensis* or *L. (L.) amazonensis* which demonstrated a balanced immune response and healing, have been shown to have a high prevalence of MHC class I restricted CD8+ T cells by immunocytochemistry analysis [11, 12]. Hence, variations at *HLA* class I and II loci, with resultant variability in interactions with parasite antigen, are likely to affect susceptibility to leishmaniasis [4].

Knowledge on the role of *HLA* genes as determinants of cutaneous leishmaniasis has mostly originated from studies in South American populations, with the predominant causative parasite species implicated in these infections being *L. braziliensis* [6, 13–16]. It has been shown that several class I (*HLA-B*) and class II (*HLA-DQ*) alleles are associated with susceptibility or protection to LCL in Venezuelan patients [13]. In a study undertaken on Mexican Mestizos, DRB1*0407, DPA1*0401 and DPB1*0101 were reported to be risk alleles while DPB1*0401 and DR2 were protective towards LCL [15]. More recently, a study conducted in southern Brazil reported a trend towards susceptibility to cutaneous lesions for several class 1 (*HLA-B)* and class II (*HLA-DRB1)* alleles while another *HLA-B* allele tended to be protective [16]. A single study on patients of South East Asian origin with cutaneous leishmaniasis due to *Leishmania (V.) guyanensis* reported a decrease of HLA-Cw7 antigen [17].

In contrast, the focus on South Asian populations has mainly been on the visceral form of the disease [18]. Leishmaniasis in Sri Lanka, until recently has been exclusively of the localised cutaneous type with over 2500 cases being diagnosed during the last 10 years [19] with numbers reported to the health system considered to be largely under-represented [20]. The causative parasite of both cutaneous and visceral disease has been identified as *L. donovani* MON 37; a zymodeme very closely related to *L. donovani* MON 2 which causes visceral disease in neighbouring India [21, 22]. However, LCL continues to be the predominant phenotype in the island in both known and new foci [19, 23, 24].

Identifying human genetic factors which operate in diverse backgrounds of parasite species and host ethnicity would contribute to understanding the complex pathogenic mechanisms underlying leishmaniasis and help identify common molecular pathways for treatment and control. We have earlier reported the observation that LCL in Sri Lanka appears to more commonly affect those of Sinhalese ethnicity [25]. A previous study by us which investigated selected polymorphisms of *TNF*, *LTA* and *SLC11A1* genes did not show any association with LCL in this population [26]. The aim of this case control study was to investigate the association of *HLA* class I/II loci with LCL in the local Sri Lankan population. We also carried out a systematic review of published literature to document all similar associations reported in other geographical regions and compared it with our findings.

## Methods

### Study population and DNA samples

The study was carried out using an already established DNA resource for conducting host genetic studies on leishmaniasis in Sri Lanka. Briefly, the study population comprised 200 unrelated patients with a laboratory confirmed diagnosis of LCL and 200 healthy, unrelated controls matched for age, gender and ethnicity. The patients were recruited at the referral clinic conducted by the Centre for Training, Research and Diagnosis of Leishmaniasis of University of Colombo and were from 11 districts of Sri Lanka; with the highest numbers from Matara and Hambantota districts in the Southern province followed by Anuradhapura district in the North Central province. Controls were recruited from the same endemic regions during the same time period as the patients. Controls were excluded if they had a previous history or diagnostic evaluation suggestive of LCL. All subjects were Sinhalese. Genomic DNA was extracted from peripheral blood lymphocytes using QIAamp DNA Mini Kit (Qiagen, USA) according to the manufacturer’s instructions. Recruitment of participants and establishment of the DNA resource has been described before [25].

### *HLA* typing

*HLA-DQB1* alleles were determined by a PCR sequence-specific priming (SSP) technique using *AllSet* Gold SSP low resolution kit according to the specifications of the manufacturer (Invitrogen, USA). PCR products were analyzed on a 2.0 % agarose gel stained with ethidium bromide (0.5 g/ml) for specific amplification patterns and alleles were assigned with SSP UniMatch (v5.1) software. Genotyping was performed at the Human Genetics Unit, Faculty of Medicine, University of Colombo. Exons 2 to 3 of *HLA-A* and *HLA-B* genes and Exons 2 to 4 of *HLA-DRB1* gene were sequenced in a subset of 140 each of patients and controls, on an Ion Proton next generation sequencing platform at a commercial sequencing laboratory [27]. The *HLA* alleles were analyzed and assigned at the same facility, by aligning against reference sequences of IMGT/HLA database and using an in house protocol adapted from published methods [28].

### Construction of a SNP panel of the HLA region

The variant calls derived from sequencing reads were used to construct a discovery SNP panel for cases and controls. All bi allelic SNPs identified in any of the subjects were extracted and included in the panel. Each individual sequence was analysed for the presence of the SNPs in the panel and zygosity at each SNP position was determined accordingly using custom built scripts. Further to this, variants within the HLA region on chromosome 6 from 6_29910603 to 6_31324183 were imputed in our discovery panel using IMPUTE2 (version2.3.1) [29] and an integrated dataset from 1000 Genomes Phase 1 [30, 31] as the reference panel.

### Statistical analysis

*HLA* allele and haplotype frequencies were estimated by the maximum likelihood method, using the Arlequin (version 3.5) software [32]. Exact tests of Hardy-Weinberg equilibrium for each locus were performed using the same. The frequencies of multi locus haplotypes were estimated under an unknown gametic phase.

The distribution of these alleles and haplotypes was compared with Pearson *χ*^2^ test with Yates correction and Fisher exact test with SPSS (version 15.0). The resulting *P* values were corrected for multiple comparisons (*Pc*) by the false discovery rate method [33] and significance was determined at *P* < 0.05. Both adjusted and unadjusted *P* values are presented. All statistical analyses were performed at the two digit-allele group level.

Comparison of SNPs between cases and controls was carried out using PLINK [34, 35]. SNPs were tested at allelic level and genotype level under dominant, additive and recessive models of inheritance. *P* value of 0.05 adjusted for multiple testing by FDR method was considered as significant. Haplotypes were constructed for selected SNPs and also compared between cases and controls using the same software. Functional prediction of the SNPs was carried out using standard bioinformatic tools including SNPNexus [36], Mutation Taster [37] and SIFT [38] and further analysed with PDB, Structure and InterPro databases [39–41]. All genomic positions reported correspond to NCBI SNP build 137.

### Systematic review

The aim of the systematic review was to document all HLA associations reported in literature that increased susceptibility to cuteanous/mucocutaneous leishmaniasis and to correlate these findings with our results.

We searched PUBMED, Web of Science (SCI), EMBASE and CINAHL for research on HLA associations with different clinical types of leishmaniasis. All databases were searched with keywords “Leishmania*” AND “HLA”, “Leishmania*” AND “Histocompatibility” in title, keywords and abstracts. All relevant studies on human populations (or case control/family studies) were selected for further review without time or language restrictions. We used the software Endnote X7 (Thomson Reuters, Carlsbad, CA 92011, USA) to filter articles. The searches had a low specificity to not to miss any relevant articles. We read all abstracts independently, and identified key articles by consensus. Full articles were obtained for all relevant studies except on one occasion where the authors could not be contacted. The article selection was conducted according to PRISMA requirements (Additional file 1: Figure S1).

## Results

The present study investigated a potential association between LCL and class I and II *HLA* loci in a South Asian population by comparing the distribution of *HLA-A*, *HLA-B*, *HLA-DRB1* and *HLA-DQB1* alleles in patients and healthy controls. The analysis was complemented by comparing the distribution of SNPs in the above *HLA* gene regions between the two groups.

### Clinical characteristics of patients

A majority of patients presented with painless, single and dry ulcers which had been present for less than 6 months. While 20 % of patients had multiple lesions, 5.5 % of patients had other affected family members. The characteristics of the patients have been described in detail previously [25] and demographic details are summarized in Table 1.

**Table 1:** Summary of demographic features of patients with limited Cutaneous Leishmaniasis

Characteristic	Number and (%) of patients
Gender	
Male	143 (71.5)
Female	57 (28.5)
Age group (years)	
0–9	3 (1.5)
10–19	28 (14.0)
20–29	48 (24.0)
30–39	58 (29.0)
40–49	27 (13.5)
50–59	15 (7.5)
60 or greater	21 (10.5)
Ethnic group	
Sinhalese	200 (100.0)
Area of residence	
Southern province	126 (63.0)
North Central province	40 (20.0)
Occupation	
Military personnel	56 (28.0)

### Distribution of HLA alleles

The frequencies of all identified *HLA* (*A, B, DRB1* and *DQB1*) alleles in patients with LCL and the control group are listed in Table 2. B*07 followed by B*40 were the two most common *HLA-B* alleles in both patients and controls out of 10 alleles which were common to both groups. At the *DRB1* locus DRB1*15 was the only allele seen in patients while 7 alleles were seen in controls with DRB1*15 being present in the majority. The *DQB1* alleles showed a comparable distribution in patients and controls except for the absence of DQB1*04 in patients. Differences in distribution were observed in several alleles with B*07 (25 % vs 48 %; *P* = 0.007; *Pc =* 0.13; OR = 0.36; 95 % CI = 0.17–0.77) more frequent in controls and DRB1*15 (100.0 % vs 76.0 %; *P* = 0.002; *Pc* = 0.01) that was over represented in patients. Overall, successful allele identification could not be performed for many samples in both control and patient groups due to some regions of DNA in the samples being refractory to amplification. Especially the number of alleles identified for *HLA-A* was limited and therefore it is not possible to draw conclusions about *HLA-A* allele distribution in cases and controls.

**Table 2:** *HLA* class I and class II allele distribution in patients with localised cutaneous leishmaniasis and population controls (n- 140 patients and 140 controls)

	Allele frequency				
Allele	Patients	Controls	*P* value^*^	*Pc* ^**^	OR (95 % CI)
*HLA-A* ^a^	2n = 18	2n = 62			
*A* ^a^ *02*	0.4444	0.2581	0.13	0.29	2.3 (0.77–6.84)
*A* ^a^ *03*	0.1667	0.0807	0.28	0.43	2.3 (0.49–10.64)
*A* ^a^ *11*	0.0556	0.0161	0.39	0.44	3.6 (0.21–60.41)
*A* ^a^ *23*	0.0556	0.0161	0.39	0.39	3.6 (0.21–60.41)
*A* ^a^ *24*	0.1111	0.0484	0.33	0.43	2.46 (0.38–15.99)
*A* ^a^ *29*	0.0000	0.1774	0.06	0.28	NA^a^
*A* ^a^ *31*	0.0556	0.1774	0.2	0.36	0.3 (0.03–2.27)
*A* ^a^ *33*	0.1111	0.0161	0.09	0.27	7.6 (0.65–89.5)
*A* ^a^ *68*	0.0000	0.2097	0.03	0.31	NA^a^
*HLA-B*	2n = 60	2n = 62			
*B* ^a^ *07*	0.25	0.4839	0.007	0.13	0.36 (0.17–0.77)
*B* ^a^ *08*	0.0333	0.0161	0.62	0.73	2.1 (0.19–23.83)
*B* ^a^ *14*	0.0167	0.0000	0.49	0.85	NA
*B* ^a^ *15*	0.0833	0.0484	0.44	0.83	1.8 (0.41–7.84)
*B* ^a^ *25*	0.0167	0.0000	0.49	0.78	NA
*B* ^a^ *27*	0.0333	0.0323	0.97	1.02	1.03 (0.14–7.6)
*B* ^a^ *35*	0.1167	0.0645	0.32	0.67	1.92 (0.53–6.92)
*B* ^a^ *37*	0.0167	0.0000	0.49	0.72	NA
*B* ^a^ *38*	0.0167	0.0645	0.18	1.16	0.25 (0.03–2.26)
*B* ^a^ *39*	0.0333	0.0000	0.24	1.14	NA
*B* ^a^ *40*	0.1167	0.0968	0.72	0.81	1.23 (0.39–3.91)
*B* ^a^ *42*	0.0000	0.0484	0.24	0.81	NA
*B* ^a^ *44*	0.1	0.0323	0.16	1.52	3.33 (0.64–17.22)
*B* ^a^ *51*	0.0333	0.0807	0.26	0.62	0.39 (0.07–2.11)
*B* ^a^ *52*	0.0333	0.0000	0.24	0.91	NA
*B* ^a^ *53*	0.0167	0.0000	0.49	0.67	NA
*B* ^a^ *55*	0.0167	0.0000	0.49	0.62	NA
*B* ^a^ *57*	0.0333	0.0000	0.24	0.76	NA
*B* ^a^ *58*	0.0333	0.0323	1.0	1.0	1.03 (0.14–7.59)
*HLA-DRB1*	2n = 34	2n = 88			
*DRB1* ^a^ *03*	0.0000	0.0114	1.0	1.17	NA
*DRB1* ^a^ *04*	0.0000	0.1023	0.06	0.21	NA
*DRB1* ^a^ *07*	0.0000	0.0682	0.18	0.43	NA
*DRB1* ^a^ *09*	0.0000	0.0227	1.0	1.75	NA
*DRB1* ^a^ *14*	0.0000	0.0227	1.0	1.4	NA
*DRB1* ^a^ *15*	1.0000	0.7614	0.002	0.01	NA
*DRB1* ^a^ *16*	0.0000	0.0114	1.0	1.0	NA
*HLA-DQB1*	2n = 358	2n = 346			
DQB1^a^02	0.1229	0.159	0.11	0.28	0.71 (0.46–1.08)
DQB1^a^03	0.2458	0.2139	0.2	0.34	1.25 (0.88–1.78)
DQB1^a^04	0.0000	0.0116	0.06	0.29	NA
DQB1^a^05	0.3045	0.3035	0.89	0.89	0.98 (0.71–1.35)
DQB1^a^06	0.3268	0.3121	0.62	0.78	1.08 (0.79–1.49)

2n, where n is the number of individuals successfully genotyped at each locus

^*^ *P* value was calculated by Pearson’s chi-square test or Fisher’s exact test

^**^ *P*c, corrected *P* values for the number of alleles tested, calculated using the False Discovery Rate method

^a^interpretations cannot be made due to limited sample size

### Distribution of HLA haplotypes

The distribution of specific 2 locus haplotypes is shown in Table 3. The most common haplotype was DRB1*15-DQB1*06 in both patients and controls. Haplotypes DRB1*04-DQB1*02 (0.0 % vs 1.8 %; *P* = 0.03; *Pc =* 0.09) and DRB1*07-DQB1*02 (0.0 % vs 1.8 %; *P =* 0.03; *Pc* = 0.09) were absent in controls while DRB1*15-DQB1*06 (5.8 % vs 15.2 %; *P* < 0.01 *Pc* < 0.01; OR = 0.3; 95 % CI = 0.2–.0.6) was present in a higher proportion of controls.

**Table 3:** Distribution of HLA DRB1*-DQB1* haplotypes in patients with localised cutaneous leishmaniasis and population controls

	Haplotype frequency^a^ (N)				
Haplotype	Patients	Controls	*P*	*Pc* ^b^	OR (95 % CI)
DRB1*04 DQB1*02	0.0000 (0)	0.0185 (5)	0.03	0.09	NA
DRB1*07 DQB1*02	0.0000 (0)	0.0185 (5)	0.03	0.09	NA
DRB1*15 DQB1*02	0.0000 (0)	0.0148 (3)	0.12	0.18	NA
DRB1*15 DQB1*03	0.0255 (6)	0.0277 (8)	0.56	0.56	0.73 (0.25–2.1)
DRB1*15 DQB1*05	0.0328 (8)	0.0501 (14)	0.18	0.22	0.55 (0.23–1.3)
DRB1*15 DQB1*06	0.0584 (16)	0.1519 (41)	0.00	0.00	0.3 (0.2–.0.6)

N indicates the number of patients or controls with the given haplotype

^a^Only the haplotypes present at a minimum of 0.01 % in the patients or controls are listed

^b^ *P*c, corrected *P* values for the number of alleles tested, calculated using the False Discovery Rate method

### Association analysis of SNPs in *HLA* region

The discovery SNP panel consisted of 28,489 SNPs spanning a region of 1.4 Mb on chromosome 6 extending from 6:29910603 to 6:31324183. After quality control 14 SNPs in 92 cases and 106 controls remained for analysis. None of the SNPs were associated with LCL at allelic level or genotypic level under different models of inheritance. At haplotype level, a haplotype consisting of alleles of two SNPs at 6:31324638 (rs281864595) and 6:31324643 (rs1050517) was seen to be higher in the controls (84.0 % vs; 93.3 %; *P* = 0.04). Annotation details of the SNPs which constituted the haplotype which tended to confer protection are given in Table 4. Structural analysis in relation to above, showed the amino acids implicated by above SNPs to be situated in the alpha 1 –alpha 2 domain of HLA*B which comprises its peptide binding region (Additional file 2: Figure S2).

**Table 4:** SNPs comprising the haplotype associated with protection to LCL

Position	dbSNP ID	Gene	Amino acid change	Predicted function	SIFT prediction (confidence)
6:31324638	rs281864595	*HLA-B*	Non synonymous	Coding	Damaging (low)
6:31324643	rs1050517	*HLA-B*	Synonymous	Coding	-

### Systematic review

The initial search with specified search methods yielded 601 abstracts. After removing duplicates and selecting studies that had reported on associations of HLA allele frequency in case control studies, 11 articles remained. Further four articles were excluded as they discussed visceral leishmaniasis. We have summarized the findings of these studies in Table 5. Overall, the reported alleles that purportedly increased or reduced susceptibility to cutaneous/mucocutaneous leishmaniasis remained highly heterogeneous in different populations studied.

**Table 5:** HLA associations for cutaneous/muco-cutaneous leishmaniasis from case control studies conducted to-date

Study	Location	Type of leishmaniasis	Sample size	Associations for susceptibility	Odds ratio (95 % confidence interval) or *p* value	Associations for resistance	Odds ratio (95 % confidence interval) or *p* value
Ribas-Silva et al. [16] 2013	Brazil	Cutaneous	160 patients	DRB1*13	1.66; (1.08–2.56)	B*45	*P* = 0.01
			270 controls				
				B*35	1.67 (1.08–2.29)		
				B*44	1.67 (1.05–2.64)		
				B*27	7.1111; (1.78–28.33)		
				B*49	6.4 (1.85–22.17)		
				B*52	12.61 (3.08–51.66)		
				A*02 B*44 DRB1*07 (haplotype)	*P* = 0.024		
				A*24 B*35 DRB1*01 (haplotype)	*P* = 0.024		
Olivo-Diaz et al. [15] 2004	Mexico	Cutaneous	65 patients	DRB1*0407	2.92 (1.68–5.06)	DPB1*0401	0.38 (0.21–0.67)
			100 controls				
				DPA1*0401	10.07 (1.25–80.73)	DR2 (DRB1*15 and DRB1*16)	0.14 (0.05–0.38)
				DPB1*0101	5.99 (1.98–18.16)		
				DQA*03011	2.14 (1.34–3.40)		
				DRB1*0407-DQA1*03011-DQB1*0302 (haplotype)	2.26 (1.27–4.00)		
				DRB1*0407- DQA1*03011-DQB1*0301 (haplotype)	6.94 (0.82–58.36)		
Samaranayake et al. [51] 2016	Sri Lanka	Cutaneous	200 patients	DRB1*15	*p* = 0.002, *Pc =* 0.01	A*68	*P =* 0.03; *Pc = 0.31*
			200 controls				
						B*07	*P =* 0.007; *Pc =* 0.13; OR = 0.36 (0.17–0.77
						DRB1*15-DQB1*06 (haplotype)	*P =* 0.00; *Pc* < 0.01; OR = 0.3 (0.2–.0.6)
						DRB1*07-DQB1*02 (haplotype)	*P =* 0.03; *Pc* = 0.09
						DRB1*04-DQB1*02 (haplotype)	*P =* 0.03; *Pc =* 0.09
Lara et al. [13] 1991	Venezuela	Cutaneous	Family study of patients and unaffected family members (n-24 families)	A*28	*P* = 0.0018	B*15	0.0062
				Bw22	*P* = 0.012		
				DQB1*0302	*P* = 0.036		
				Bw22CF31 (haplotype)	*P* = 0.0076		
				Bw22DR*11DQ*7 (haplotype)	*P* = 0.0163		
				DQw3	Pc = 0.036		
Petzl-Erler et al. [49] 1991	Brazil	Muco-cutanous	43 patients	DQB1*03	*p* = 0.006	DR2 (DRB1*15 and DRB1*16)	*p* = 0.004
			111 controls				
el-Mogy et al. [50] 1993^a^	Egypt	Cutaneous	27 patients	A*11	NA		
				B*5	NA		
				B*7	NA		
Barbier et al. [17] 1987	Asian refugees in French Guiana	Cutaneous	32 cases			Cw7	*p* = 0.01
			55 controls				

^a^_Full article was not available_

## Discussion

We studied the association of variations at *HLA* class I (*HLA-A*, *HLA-B*) and *HLA* class II (*HLA-DRB1*, *HLA-DQB1*) loci with susceptibility to localised cutaneous leishmaniasis in Sinhalese in Sri Lanka. The results presented in this report are, to our knowledge, the first on *HLA* associations in cutaneous leishmaniasis in a South Asian population.

The distribution of HLA alleles showed 7 alleles of *HLA-A*, 18 alleles of *HLA-B* and 4 alleles of *HLA-DQB1* to be present in patients with LCL. Notably, the *HLA-B* locus was more polymorphic in the patients than in the control population. Eventhough only HLA-DRB1*15 allele was observed in the patients, this is likely to be due to the limited sample number, as evident by the polymorphic nature of the locus in the larger control group. The overall distribution of alleles in the controls conforms to previous reports from the country [42] and adds to the limited data available on *HLA* polymorphisms in this population.

The case control analysis did not show any statistically significant associations between *HLA* markers and LCL after correcting for multiple testing. However, comparisons of trends observed in this study with findings from other populations are noteworthy. For instance, the allele frequency of HLA-B*07 (*P* = 0.007; *Pc =* 0.13; OR = 0.36; 95 % CI = 0.17–0.77) was higher in controls suggesting a protective tendency by this allele. Interestingly *HLA Bw22*, which is similar to HLA-B*07 in the amino acid sequence at alpha 1-helix [43], has been shown to be a risk factor for cutaneous leishmaniasis due to *L. braziliensis* in Venezuelans [13]. The same study reported a protective effect conferred by *HLA-B15,* whereas the frequency of *HLA-B15* was twice that of controls in our patients. Ribas-Silva et al. [16], investigating genetic susceptibility to different clinical manifestations of American cutaneous leishmaniasis in southern Brazil, reported HLA B*35 and B*44 to confer susceptibility and B*45 to confer protection against cutaneous lesions. Both HLA B*35 and B*44 were over represented in our patient group but did not reach significant proportions.

At the *HLA-DQ* locus, *DQw3* (DQB1*03) and *DQw8* (DQB1*0302) have been associated with susceptibility to LCL [13] whereas the present study showed an equivalent distribution of DQB1*03 between the two groups. In contrast to the current study where DRB1*15 (*P* = 0.002; *Pc* = 0.01) appeared to be a risk allele, DR2 serotype which encompasses DRB1*15 and DRB1*16 was found to be protective in Mexican Mestizos [15]. Further, DRB1*13 and DRB1*04 were considered risk factors in this same population and in Brazilians [16] respectively.

The small sample number is a limitation of this study. This may lead to a bias in estimating allele and haplotype frequencies and thus caution should be exercised in extrapolating them to the study population. This may also explain some of the observations made, for instance the patient group being monomorphic at the *HLA-DRB1* locus, and calls for replication of findings in a bigger cohort in validating the significant associations involving this locus.

The apparently contradictory results for the same locus in different global populations as we report here, is a common occurrence in genetic association studies. These differences most probably reflect the LD patterns across the gene regions under focus, with the clinical outcome being the concerted effect of several loci, some of which may have not been considered in the particular study. This variability is further compounded by the extensive polymorphism in the *HLA* genes [44]. However, notably the only genome wide association study to be conducted on leishmaniasis reported risk variants for the visceral phenotype in the *HLA-DRB1-HLA-*DQA1 region, which were common to population groups across different geographical areas and affected by different parasite species (Fakiola et al. [18]). Further, patients with other affected family members may indicate shared living environment or a true genetic association. Thus, complimenting population based case control analyses such as that undertaken in the present study, with multi-case family based linkage analyses and transmission disequilibrium testing would also strengthen the findings.

The systematic review showed that there are only a handful of studies that have assessed the *HLA* polymorphism in cases and controls worldwide. Most of these studies are from Latin America and our study in fact is the first from Asia on cutaneous leishmaniasis. There are several studies that have assessed *HLA* polymorphism in visceral leishmaniasis including a large scale multicentre study in Brazil and India [45], but not for cutaneous leishmaniasis. It is also noted that while there are two studies that explicitly stated no *HLA* associations for visceral leishmaniasis (out of 4 studies) when compared with controls [46, 47], all studies that assessed the same for cutaneous/muco-cutaneous leishmaniasis report otherwise. There can be reporting bias with non-publication of negative results but even in the two studies that showed a significant association it was just limited to 3 *HLA* alleles in both studies combined [45, 48]. The synthesis of observations (Table 5) shows that the purported susceptibility or protection gained by certain HLA alleles or haplotypes has rarely been independently verified by subsequent studies except for few exceptions (DR2 serotype). There can be several reasons for this. Firstly, the studies are few in number and small in sample size. Secondly, they are spread across different countries/geographical regions that have populations isolated from each other with plausibly different baseline HLA allele frequencies in the population. Thirdly, the *Leishmania* species (hence antigens) in each of these regions differ which can lead to heterogeneity in host response. Overall, it raises the importance of screening individual populations locally to identify protective and susceptible characteristics of host genome which would be important to design effective vaccines for local populations.

## Conclusions

Overall, several of the alleles in the *HLA-B* region which have been associated with susceptibility or protection towards cutaneous leishmaniasis in other populations, were also shown to differ between patients and controls of our study group. The two SNPs on *HLA-B* gene, which result in a haplotype found to be more frequent in controls as shown by the complementary SNP analysis, have not been widely reported to aid definitive conclusions. Nevertheless, the fact that these implicate two adjacent amino acids in the alpha1 – alpha2 region of HLA*B which comprises its antigen recognition region, suggests candidate loci to be considered in expanded work in this study group.

Further studies in this population, on larger sample numbers conferring better statistical power will be required to support the findings of this study. The findings should also be complemented with higher resolution HLA typing, high density SNP analyses of the HLA region and also functional studies on interactions between host immune response cells and parasite antigens in a HLA restricted setting.

## Abbreviations

HLA, human leucocyte antigen; LCL, localized cutaneous leishmaniasis; PRISMA, preferred reporting items for systematic reviews and meta-analyses

## Additional files

Additional file 1: Figure S1. PRISMA flow chart for systematic review. (DOCX 76 kb) Additional file 2: Figure S2. Three dimensional model of HLA-B protein based on PDB entry 1HSA (DOI:10.2210/pdb1hsa/pdb). Amino acids at positions 55 and 57 in the alpha1 and alpha2 domains of the molecule which constitute the antigen recognition region are highlighted. (TIF 376 kb)
