# Supplementary figures and images for: Association of HLA class I and II genes with cutaneous leishmaniasis: a case control study from Sri Lanka and a systematic review
Source: BMC Infect Dis. 2016 Jun 14;16:292. doi: 10.1186/s12879-016-1626-8 (PMC4908677; doi:10.1186/s12879-016-1626-8)

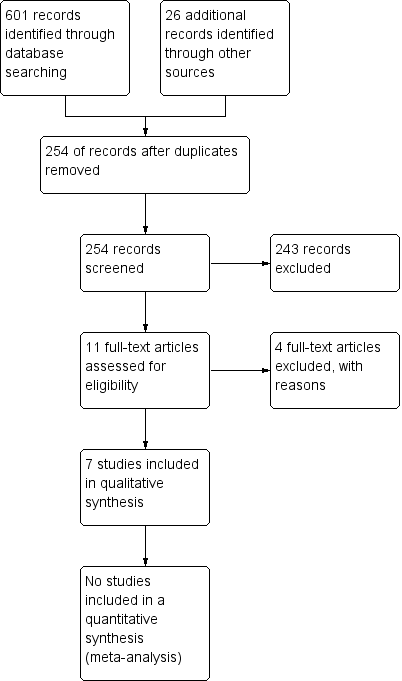

Supplement: Additional file 1: Figure S1. — PRISMA flow chart for systematic review. (DOCX 76 kb) [file 12879_2016_1626_MOESM1_ESM.docx]

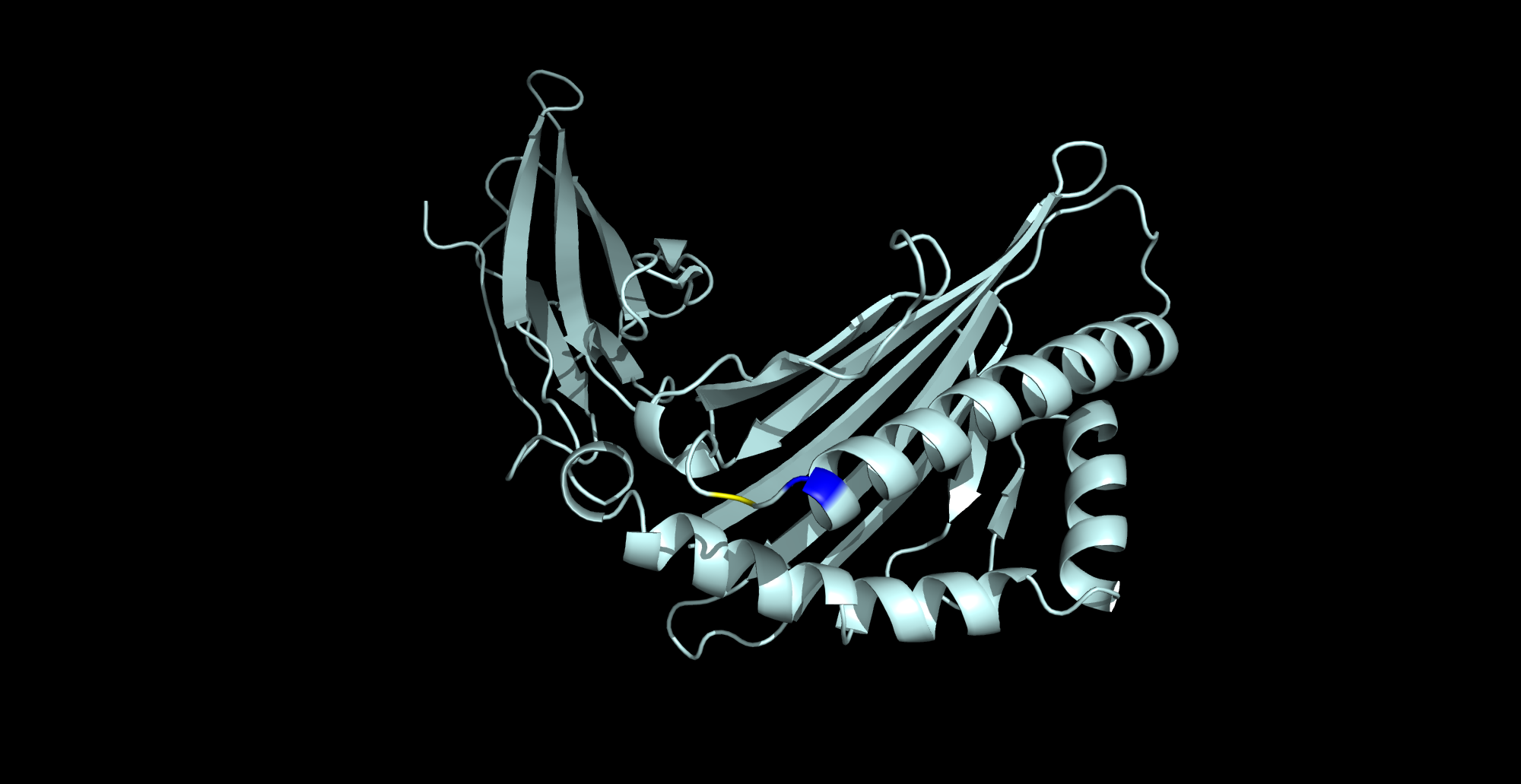

Supplement: Additional file 2: Figure S2. — Three dimensional model of HLA-B protein based on PDB entry 1HSA (DOI:10.2210/pdb1hsa/pdb). Amino acids at positions 55 and 57 in the alpha1 and alpha2 domains of the molecule which constitute the antigen recognition region are highlighted. (TIF 376 kb) [file 12879_2016_1626_MOESM2_ESM.tif]
